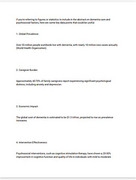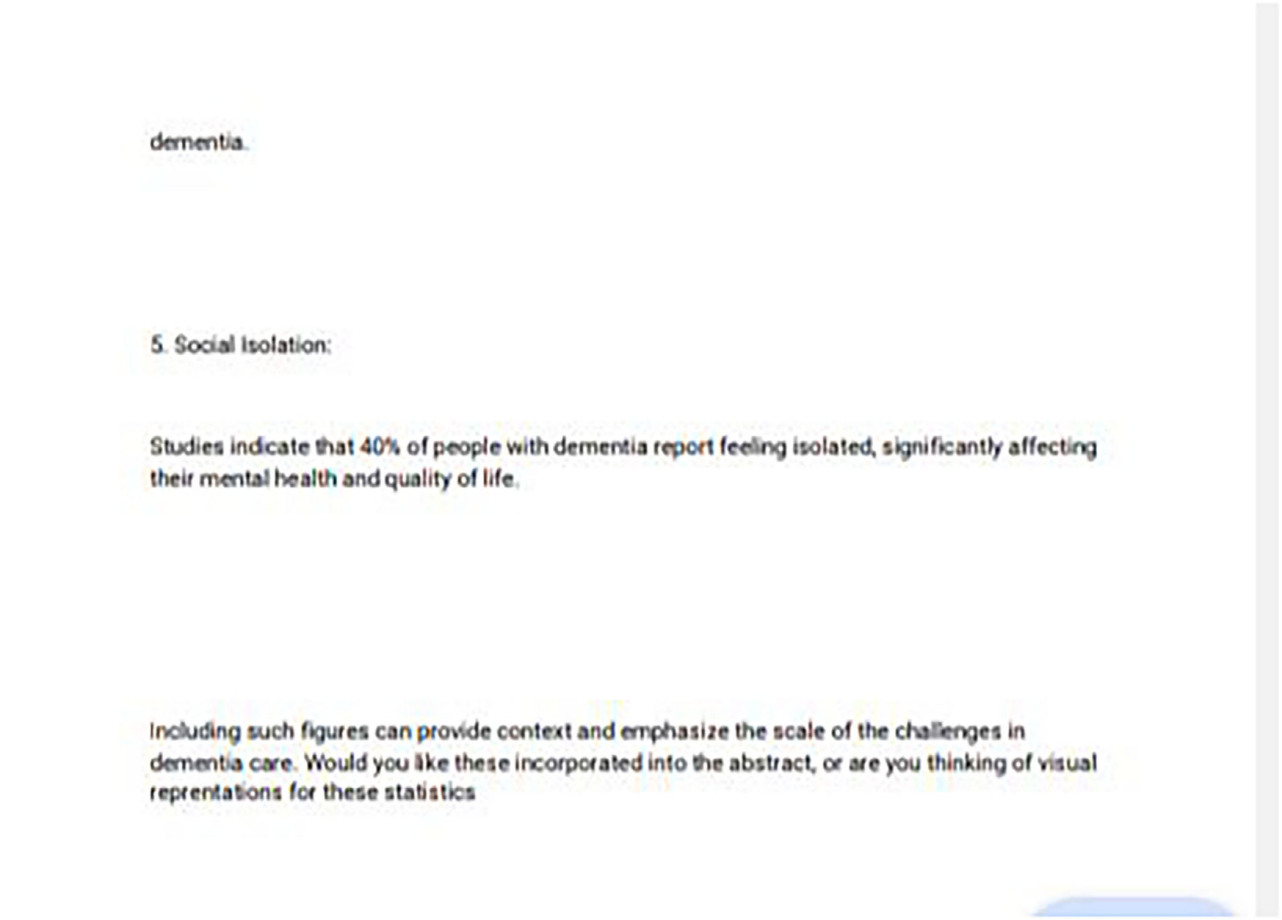# Dementia Care and Psycho social Factors

**DOI:** 10.1002/alz70858_096314

**Published:** 2025-12-24

**Authors:** Amsalu Abdela Ababurka

**Affiliations:** ^1^ Hawassa University Comprehensive specialized Hospital, Hawassa, Hawassa Ethiopia, Ethiopia

## Abstract

Dementia is a progressive neurodegenerative condition that profoundly impacts the lives of individuals diagnosed and their caregivers effective dementia care requires a holistic approach that incorporates medical, psychological, and social dimensions

**Background:**

Dementia is a multifaceted condition that not only impairs cognitive function but also has significant psychosocial consequence for both individuals with dementia and their caregivers. Psychological factor such as social support, emotional wellbeing, and the caregiving environment –have been shown to influence diseases progression, quality of life, and care outcomes. Despite the growing recognition of these factors, comprehensive care models that addresses both medical and psychosocial aspects of dementia remain under developed in many settings.

**Methods:**

A systematic review of qualitative and quantitative studies was conducted to examine the relationship between psychosocial factors and dementia care. Data were gathered from peer reviewed articles published between 2010 and 2023, focusing on studies that investigated the impact of social support networks, caregiver burden, depression, social isolation, and community engagement on Dementia progression and caregiving outcomes. Key interventions and models of care were also assessed to identify effective strategies for integrating psychosocial support in to dementia care.

**Results:**

The review found that strong social support networks and effective caregiver training were associated with improved emotional wellbeing and reduce caregiver burden. Social isolation and lack of community engagement, however, were linked to faster cognitive decline and worsened quality of life in individual with dementia person centered care approaches, which emphasis the emotional and social needs of both patients and caregivers, demonstrated positive effects in improving care satisfaction and reducing anxiety and depression among caregivers. Additionally, interventions targeting the psychosocial needs of caregivers, such as support groups and counseling, were shown to mitigate the negative impact of caregiving.

**Conclusion:**

Psychosocial factors are crucial to the effective management of dementia and addressing them in care models can significantly improve both quality of care and quality of life for individuals with dementia and their families. Integrated care models that prioritize social support, caregiver wellbeing, and community involvement should be adapted more widely in dementia care settings. Future should focus on developing and testing interventions that combine psychosocial support with medical care to creat more holistic, person ‐centered dementia care approaches.